# Effects of *Urtica urens* in the Feed of Broilers on Performances, Digestibility, Carcass Characteristics and Blood Parameters

**DOI:** 10.3390/ani13132092

**Published:** 2023-06-24

**Authors:** José Teixeira, Pedro Nunes, Divanildo Outor-Monteiro, José Luís Mourão, Anabela Alves, Victor Pinheiro

**Affiliations:** 1 Department Animal Science, University of Trás-os-Montes e Alto Douro, 5000 Vila Real, Portugal; pedronunes@utad.pt (P.N.); divanildo@utad.pt (D.O.-M.); jlmourao@utad.pt (J.L.M.); aalves@utad.pt (A.A.); vpinheir@utad.pt (V.P.); 2 Animal and Veterinary Research Centre (CECAV), University of Trás-os-Montes and Alto Douro, 5000 Vila Real, Portugal; 3 Al4Animals, Department of Animal Science, University of Trás-os-Montes and Alto Douro, 5000 Vila Real, Portugal; 4 Department Veterinary Science, University of Trás-os-Montes e Alto Douro, 5000 Vila Real, Portugal

**Keywords:** *Urtica urens*, broilers, performances, digestive tract, blood parameters, carcass

## Abstract

**Simple Summary:**

*Urtica urens* used as an additive in animal feed may have antibacterial, antioxidant, and anti-inflammatory properties. Some studies suggest a positive effect of *Urtica urens* on the immune system, digestive tract development, regulation of appetite and voluntary feed intake, stimulation of basal metabolic rate and enhancement of meat quality. In a broiler trial, an increase in growth performance and a decrease in HDL (high-density lipoprotein) cholesterol were observed. The results suggest that the use of *Urtica urens* can improve broiler health and performance.

**Abstract:**

With the aim of improving animal health and productivity, plants or plant extracts that have antimicrobial, antifungal and antioxidant properties are often used in studies with broilers. The aim of this work was to investigate the effect of *Urtica urens* in broilers. Ninety male Ross chicks were used, randomly placed in 30 pens (three broilers per pen). The broilers were assigned to three treatment groups: group CT (control) received a basal diet; group UU1 received a basal diet with 1% dried *Urtica urens*; and group UU2 received a basal diet with 2% dried *Urtica urens*. Each treatment consisted of two feeds, distributed from day 1 to 15 (starter) and from day 15 to 36 (grower). As a result of this study, broilers in the UU1 treatment group had higher weight on day 15 (*p* = 0.029) and day 36 (*p* = 0.014) than those in treatments CT and UU2 and a higher daily weight gain between days 1 and 15 (*p* = 0.028) and days 1 and 36 (*p* = 0.014). Broilers in the UU1 and UU2 groups had lower serum HDL cholesterol (88.8 and 88.9 mg/dL, respectively) than the CT (96.1 mg/dL). In conclusion, the use of dried *Urtica urens* at 1% as an additive in broiler diets may affect growth performance and blood HDL cholesterol.

## 1. Introduction

The demand to improve animal health and efficiency has led to research into new additives to replace the use of antibiotics as growth promoters. The use of plants, such as *Urtica urens*, has been tested because they may have antimicrobial, antifungal and antioxidant properties [1]. Some studies show that some nettle varieties can improve the health, nutrition and environmental resilience of livestock production [2] and make animals more resistant to bacterial infections [3]. However, the exact mode of action of these plants and their bioactive compounds affecting broiler performance is not well understood [4]. The species of the stinging nettle family (*Urticaceae*) are native to Europe and parts of western Asia. Some studies have attributed nutritional properties and functional biological activities to the stinging nettle [5], and it is considered an interesting component of animal feed [6]. Its effects on performance and health have been studied in quail [7], rabbits [8,9], rats [10], farmed fish [11] and broilers [12]. In the aerial parts of *Urtica urens*, the main components are lipids and proteins [13], like glutamic acid, aspartic acid, alanine, and leucine, which are the major amino acids in *Urtica urens* and *Urtica dioica* [14]. The main polyphenols are caffeic acid, p-coumaric acid, ferulic acid, sinapic acid (total phenolic content = 0.8 g/100 g), C-glycosyl flavonoids and O-glycosyl flavonoids and are responsible for the anti-radical and antioxidant capacity (DPPH = 10.60 TE) [15]. The *Urtica dioica* is the most studied nettle species and is known for its antioxidant and anti-inflammatory properties [12,16,17]. Some studies on the antioxidant activity of *Urtica dioica* have shown that the dried leaves have higher antioxidant activity than the fresh leaves [17]. According to [13], chlorogenic acid is the main component of *Urtica dioica* responsible for antioxidant activity, and according to [18], this is due to phenolic compounds, especially hesperidin. These active compounds in stinging nettle have been associated with improved weight gain and feed conversion in broilers [19], reduced health risks [20,21,22,23] and improved performance [24]. The use of stinging nettles in broilers had positive effects on meat quality [4], performance, carcass characteristics, blood biochemical parameters [25] and cholesterol reduction [26]. In the diet of broilers, nettles play a positive role in maintaining a balanced microflora in the digestive tract [27]. The aim of this work was to investigate the effect of *Urtica urens* in broiler diets on growth performance, blood composition, and carcass and digestive tract characteristics, because limited information is available on dried *Urtica urens*.

## 2. Materials and Methods

All the procedures of the experiment were in accordance with the guidelines and regulations of the Organism for Animal Welfare of the University (ORBEA; process number 1899-e-DZ-2021), in compliance with the provisions of Legislative Decree No. 113/2013 of 7 August, which transposed into Portuguese law the Directive No. 2010/63/EU on the protection of animals used for scientific purposes.

### 2.1. Collecting and Preparation of the Urtica urens

Whole *Urtica urens* plants were harvested at the end of the vegetative cycle in December, and the aerial parts of the plants were collected and then dried at 50 °C in an oven with forced ventilation for three days. After drying, the stalks and leaves of the plants were ground and filtered through a 1 mm sieve to obtain the dried *Urtica urens*.

### 2.2. Animals and Experimental Design

The experiment was conducted at the University of Trás-os-Montes and Alto Douro in Portugal. A total of 90 one-day-old male chicks (Ross) were used. The broilers were randomly housed in 30 pens of 0.30 m^2^. In each pen, 3 broilers were housed. Each pen had two feeders and one drinker. Wood chips were used as litter material (3.8 kg/m^2^). The chicks were subjected to 3 treatments (10 pens and 30 birds per treatment): the control treatment with the basic feed (CT) and treatments UU1 and UU2 with the inclusion of 1% and 2% dried *Urtica urens*, respectively. In each treatment, 2 different feeds were used according to the age of the broilers, the starter feed from day 1 to day 15 and the grower feed from day 15 to day 36 (Table 1). Feed and water were distributed ad libitum.

### 2.3. Growth Performances

Animals were weighed individually on the 1st, 9th, 15th, 27th and 36th day of life, and feed intake was monitored. Daily weight gain, daily feed intake and feed conversion ratio were calculated from these data.

### 2.4. Chemical Analysis

All samples (feed and feces) were dried at 50 °C to a constant weight in a forced-air oven (Venticell, MMM Group, Munich, Germany). Samples were ground over a 1 mm sieve (Tecator Cyclotec 1093 Sample Mill, Foss SA, Sweden) and prepared for chemical analysis. AOAC [29] procedures were used to determine dry matter (DM) (No. 934.01), organic matter (OM) and crude ash (No. 942.05) as well as ether extract (EE, No. 920.39) and total nitrogen (N) using the Kjeldahl method (954.01). The crude protein (CP) content was calculated as N × 6.25, according to Kjeldahl. Neutral detergent fiber (NDF) content was determined without using sodium sulfite and α-amylase, according to the methods proposed by Pigden et al. [30] and Van Soest et al. [31].

### 2.5. Slaughter, Intestinal Tract and Carcass Characteristics

At the end of the growth period (36 days), 30 broilers (10 per treatment) were slaughtered via cervical dislocation and bleeding was carried out immediately after. After slaughter, the feathers were removed and the chickens were eviscerated. Carcasses were weighed two hours after slaughter to determine the carcass yield.

The digestive tract, liver, gizzard, caecum, duodenum and ileum were weighed; the length of the caecum, duodenum and ileum was measured; and the color of the liver was determined using a Minolta Chroma Meter CR-200 colorimeter (Minolta Camera, Osaka, Japan).

The colorimeter was used to measure the color of the skin and breast muscle after 24 h of chilling. The color attributes referred to as Tom-Hue (H*) and Chroma (C*) were measured according to Leite et al. [32]. At the same time breast muscle pH was measured with a digital potentiometer.

### 2.6. Blood Analysis

At the time of slaughter, 4 mL of blood was collected from the jugular vein of each broiler. Serum was collected through sedimentation, and samples were analyzed for total cholesterol, high-density lipoprotein (HDL), low-density lipoprotein (LDL), triacylglycerol (TAG), glucose, total protein, albumin, globulin, total bilirubin, uric acid, alanine transaminase (ALT), aspartate transaminase (AST), alkaline phosphatase (ALP) and gamma-glutamyl transferase (GGT), according to the method described by Rosa et al. [33].

### 2.7. Caecal Volatile Fatty Acids Analysis

Samples of caecal content from each broiler collected after slaughter were stored at −20 °C until VFA analysis. Samples were diluted 1:5 in distilled water and centrifuged at rpm for 15 min. VFA analysis was performed using a SHIMADZU gas chromatograph (model GC 141 B, Kyoto, Japan), equipped with a flame ionization detector and a SUPELCO capillary column (Nukol, 30 m × 25 mm). VFA analysis was performed following the method of J. W. Czerkawski [34].

### 2.8. Villis Development

A sample of the last section of the ileum (10 cm) was taken from each slaughtered broiler and fixed in 10% buffered formaldehyde, then trimmed and processed for routine histologic assessment. After sectioning (3 microsections), slides were stained with H&E, using the protocol described by Mourão et al. [35].

**Figure 1:**
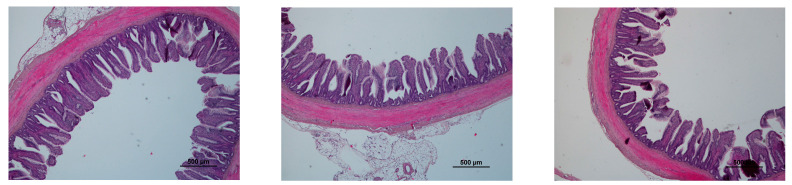
Three images of an ileum sample.

Three images were taken of each sample (Figure 1) and five villi were measured on each image (fifteen measured villi per sample). 

The measurements obtained on these images were villi height (VH), villi width at two points in a high position (at the top of the villi) and in a low position (at the crypt) and crypt depth (CD). Using these measurements, the villus absorption area (***aa***) was calculated according to the equation below, where ***r*** is the radius and ***l*** is the length of the villus.

$$aa=\frac{4\pi r^{2}}{2}+2\pi rl$$

### 2.9. Statistical Analysis

Statistical analysis was performed with linear regression models, considering treatment as a factor of variation. The comparison of means was performed via Tukey’s test to identify the differences between treatments. All data were analyzed using the JMP^®^ Pro 16.2.0 program. The significance level used was 5%.

## 3. Results

### 3.1. Growth Performances

Table 2 shows the growth performances of broilers. No differences were found in the initial weight of the groups, but there were significant differences on the 15th and 36th day of life, with the UU1 broilers having a higher live weight (about 170 g after 36 days) than the broilers in the other two treatments (*p* < 0.05). Daily weight gain was higher in the broilers of the UU1 treatment than in the broilers of the control and UU2 treatments in the period from 1 to 15 days (*p* = 0.028) and in the whole period (*p* = 0.014). Daily feed intake and feed conversion were not affected by the feed treatments. However, daily feed intake showed a trend toward higher consumption when 1% nettle was added to the diet.

### 3.2. Digestive Tracts Characteristics

Table 3 shows the effects of the treatments on digestive tract weight, length and liver color of broilers. No effects were observed on any of the parameters measured.

### 3.3. Carcass Characteristics

Table 4 shows the effects of the treatments on carcass characteristics. There are differences in the luminosity attributes (L*) of the breast muscle (*p* < 0.046), showing higher values in the UU2 treatment group than in the CT and UU1 treatment groups. There were no differences in the other carcass parameter measurements.

### 3.4. Blood Composition

The broilers in the CT treatment group had significantly higher HDL cholesterol content than those in treatment groups UU1 and UU2 (about 7.6%), which did not differ from each other (Table 5). The lowest value of total cholesterol and LDL was also observed in the UU1 treatment, but only a trend toward a difference was observed.

### 3.5. Caecal VFA

The VFA concentration and the proportion of major volatile fatty acids in the cecal content at an age of day 36 are shown in Table 6. Acetic acid was the predominant VFA, followed by butyric acid and propionic acid. No significant differences were observed between treatments when *Urtica urens* was added to the diet.

### 3.6. Villi Development

Ileal villi were measured in slaughtered broilers (Table 7). The results of these histological analyses showed no significant differences between treatments.

## 4. Discussion

The results suggest that broilers fed diets with 1% dried *Urtica urens* have better growth performance when compared with the control group and the group fed with 2% dried *Urtica urens*. Differences were found in live weight and daily weight gain. Similar results were observed in [36], where positive differences were found in weight gain and feed intake with the 1% nettle treatment. However, the UU2 treatment had no significant effects. Another study [37] reported that the addition of 2% nettle in broiler diets had positive effects on body weight gain, average daily gain, daily feed intake and feed conversion ratio. In terms of daily feed intake, the UU1 group ate 4.0 g/d more than the others, but no significant differences were found between treatments, as in other works [19,38]. However, [39] reports that the addition of 0.5% nettle significantly decreased feed intake (*p* < 0.05). Another study [40] reported that the higher the percentage of nettles incorporated, the lower the performances, live weight, feed intake and higher feed conversion ratio values, which could explain why the UU2 treatment has worse performances than UU1. In regard to FCR, no differences were found between the treatments, and the UU2 treatment showed the lowest results. This result was also observed in [41]. However, in [19], when nettles were incorporated into broiler diets at 1% and 1.5%, it significantly (*p* < 0.05) improved FCR, and [20] found differences (*p* = 0.04) in the 1.5% nettle treatment group (1.46) in comparison to the controls (1.84). It is likely that the different results of our work compared to others are due to the use of different diet compositions, different nettle incorporations, different nettle species used and different broiler strains.

No differences were found in the weight and length of the digestive tract. The same result was reported in [40]. In terms of liver color, we found no differences between the treatments. In disagreement with our study, [38] found the highest value in liver color b*, in broilers supplemented with nettles.

No effects were observed in carcass yield, which agrees with [23,38], who reported that nettle supplements did not influence this parameter. Furthermore, regarding the different levels of nettle incorporation, [39,40] used 5% and [41] used 0.75 and 1.5% and reported that these levels significantly affected carcass characteristics. With a 9% incorporation of nettles, [42] obtained a significantly higher carcass yield (68%) when compared to the control (65.4%).

In the pH of the breast muscle, no differences were found between the groups. In regard to the color of the beast muscle, differences in skin L * (over 2.3 and 2.6 units) were found when compared to UU1 and the control. This could be an indicator associated with lower ultimate pH and poorer water-holding [43]. In terms of the skin carcass color, no effects were observed; however, [38] related differences in skin b * (*p* < 0.05) and reported that this was massively intensified by the addition of nettles to broiler feed. According to [44], this may have been caused by the carotenoids lutein and β-carotene present in nettles. The lack of significant effects observed in skin color may be due to the rate of nettle incorporation into broiler feed [45].

Our findings revealed that *Urtica urens* significantly lowered HDL cholesterol and tended to lower the LDL and total cholesterol in broilers. Consistent with our findings, [20,23,36] observed that the inclusion of nettles in the diet of broilers reduced blood cholesterol. In disagreement, Khosravi et al. [25] observed that the incorporation of 1 g/kg of nettles had no effect on blood cholesterol (but used nettle extract), and the same tendency was seen in [46], with an incorporation of a higher level of nettles (5%), and in [41], with the use of different levels of nettles in the starter and grower broiler feed.

In regard to the other parameters of blood composition, no differences were found. In [40], these were not affected by dietary treatment with nettles. However, [20] reported that total cholesterol and triglycerides were significantly reduced by diet with 1.5% nettle incorporation. Regarding liver function, [47] determined that several hepatic enzymes in serum, such as ALT, AST, ALP, γ-glutamyl transpeptidase (γ-GGTP) and lactate dehydrogenase (LDL), could be used to assess the functional state of the liver and to detect liver injury. In our study, no differences were found between treatments, and the same results were obtained in [46], regarding the hepatic enzyme ALT. Uric acid is synthesized in the liver [48], and significant increases in uric acid levels are indicative of nephrotoxicity in broiler chickens [49], whose mean values are between 25 to 45 g/L [50]. Our results found no differences between treatments, perhaps because uric acid production depends on dietary protein intake and the breakdown of endogenous purines through xanthine oxidase [51].

The main VFAs, i.e., acetic, propionic, and butyric, have a bacteriostatic effect [52], and they are also responsible for reducing the number of *Enterobacteriaceae* in the cecum of broilers during growth. In the present study, we found no differences in volatile fatty acids, and no trials with broilers fed *Urtica urens* in which VFA was measured were found in the literature. However, in lambs fed with *Urtica cannabina*, there were higher amounts of butyrate, propionate, and total VFA, which resulted in a significant increase in the width of papillae on both sides of the rumen [53]. However, in [9], a trial using nettle-fed rabbits did not influence the amount of VFA.

Increased villus height ensures increased digestive and absorptive functions by increasing the apparent surface area of the villi [54], thus providing a larger absorptive area and consequently increased feed digestion capacity [55]. This increase in villus development may be related to its protective barrier function against toxic substances, such as polyphenolic compounds [56]. Increased villus growth in the gut enhances the development of beneficial microorganisms, modulates the immune system and increases the animal’s ability to resist intestinal disturbances [57]. In our study, considering the results of the villi measurements of the ileum, we verified that there were no significant differences between treatments.

## 5. Conclusions

In conclusion, the use of dried *Urtica urens* in broiler feed, at a level of 1% of incorporation, improves growth performance and decreases blood HDL cholesterol. In the future, it will be important to work with other incorporation levels and evaluate other parameters that will give us an indication of the true value of nettles as an additive in broiler feed.

## Figures and Tables

**Table 1:** Ingredients and chemical composition of the experimental diets and *Urtica urens*.

			Starter			Grower	
	*U. urens*	Control	UU1	UU2	Control	UU1	UU2
Ingredients (g/kg as fed)							
Nettle (*Urtica urens*)		-	10.00	20.0	-	10.00	20.0
Corn		500	495	490	670	663	657
Triticale		120	119	118	-	-	-
Soybean meal		320	317	314	280	277	274
Animal fat		15.0	14.8	14.7	25.0	24.8	24.5
Sunflower meal		20.0	19.8	19.6	-	-	-
Calcium		9.00	8.91	8.82	8.20	8.12	8.03
Sodium		1.40	1.38	1.37	1.30	1.28	1.27
Fatty acids		4.60	4.56	4.51	5.50	5.45	5.40
Additives ^1^		10.00	9.90	9.80	10.00	9.90	9.80
Chemical composition analyzed (g/kg DM)							
Organic matter	802	936	923	921	944	945	947
Crude protein	224	217	221	224	198	203	210
NDF	309	115	117	119	137	140	144
Crude fat	56	41	43	48	54	53	51
ADF	249	46	48	52	48	49	53
ADL	46	6	6	7	8	8	9
ME (MJ/kg DM) ^2^	-	12.09	12.21	12.35	12.73	12.70	12.68

^1^ The following were provided per kg of diet: vitamins—vitamin A = IU, vitamin D3 = 5000 IU, vitamin E = 65 mg; compounds of trace elements—iron = 50 mg, iodine = 0.4 mg, copper = 17 mg, manganese = 100 mg, zinc = 37.5 mg, selenium = 0.4 mg; amino acids—L-lysine = 1994 mg, methionine = 3200 mg; antioxidants—butylhydroxytoluene (BHT) = 7.2 mg, propyl galate = 0.6 mg. ^2^ Estimated values according to the equation of Carré et al. [28].

**Table 2:** Effects of the incorporation of *Urtica urens* into the broiler feed on the growth performances of broilers at different intervals (*n* = 10 per treatment).

		Treatments			
	Control	UU1	UU2	SEM	*p*-Value
Live weight (g)					
Initial	41.8	42.0	41.6	0.50	0.885
Day 15	487 ^b^	519 ^a^	487 ^b^	11.4	0.029
Day 36	2374 ^b^	2522 ^a^	2328 ^b^	48.4	0.014
Daily weight gain (g/d)					
1–15 days	29.7 ^b^	31.8 ^a^	29.1 ^b^	0.75	0.028
15–36 days	89.9	95.4	88.1	2.23	0.056
1–36 days	64.8 ^b^	68.9 ^a^	63.5 ^b^	1.34	0.014
Daily feed intake (g/d)					
1–15 days	35.3	38.6	36.5	1.06	0.094
15–36 days	139	146	138	2.7	0.079
1–36 days	96.1	100.0	96.0	1.43	0.071
Feed conversion ratio					
1–15 days	1.22	1.24	1.26	0.034	0.672
15–36 days	1.55	1.58	1.58	0.024	0.605
1–36 days	1.49	1.49	1.51	0.024	0.704

UU1—basal diet with 1% dried *Urtica urens*; UU2—basal diet with 2% dried *Urtica urens.* SEM: standard error of the mean; mean values in the same row with different superscripts are significantly different (*p* < 0.05).

**Table 3:** Effects of incorporation of *Urtica urens* in the broilers feed on digestive tract relative weight and length, and liver color (*n* = 10 per treatment).

		Treatments			
	Control	UU1	UU2	SEM	*p*-Value
Weight (g/kg)					
Digestive tract	97.7	106	102	3.74	0.294
Digestive tract fat	19.7	21.0	18.6	0.75	0.230
Liver	17.9	18.7	18.9	0.72	0.642
Gizzard	22.9	24.4	25.3	0.96	0.235
Cecum	5.2	5.76	5.34	0.53	0.723
Duodenum	18.7	20.8	19.9	1.43	0.580
Ileum	12.6	13.7	12.7	1.05	0.724
Length (cm/kg)					
Duodenum	47.0	45.4	46.9	1.33	0.620
Ileum	32.3	33.6	32.8	0.87	0.626
Cecum	7.39	8.30	7.99	0.33	0.163
Liver color					
l*	32.9	34.5	32.7	0.59	0.393
a*	20.8	21.3	21.5	0.33	0.669
b*	17.1	20.0	17.2	0.69	0.159
Tom-Hue	0.68	0.75	0.67	0.02	0.182
chroma	27.0	29.3	27.6	0.58	0.252

UU1—basal diet with 1% dried *Urtica urens*; UU2—basal diet with 2% dried *Urtica urens.* l*: lightness; a*: redness; b*: yellowness. SEM: standard error of the mean.

**Table 4:** Effects of the incorporation of *Urtica urens* into the broiler feed on carcass measurements (*n* = 10 per treatment).

		Treatments			
	Control	UU1	UU2	SEM	*p*-Value
Carcass yield (g/100 g)	75.0	74.5	74.4	0.29	0.718
pH breast muscle	5.88	5.88	5.86	0.02	0.891
Color of breast muscle					
l*	50.2 ^b^	50.5 ^b^	52.8 ^a^	0.48	0.046
a*	1.42	1.97	1.86	0.17	0.414
b*	16.9	17.8	18	0.32	0.338
Tom-Hue	1.49	1.46	1.47	0.01	0.517
Chroma	17.0	17.9	18.1	0.32	0.287
Skin carcass color					
l*	60.3	60.1	59.1	0.45	0.519
a*	2.66	1.59	2.20	0.27	0.288
b*	28.2	27.1	26.7	0.67	0.673
Tom-Hue	1.48	1.51	1.49	0.01	0.267
Chroma	28.3	27.1	26.8	0.69	0.658

UU1—basal diet with 1% dried *Urtica urens*; UU2—basal diet with 2% dried *Urtica urens.* l*: lightness; a*: redness; b*: yellowness. SEM: standard error of the mean; mean values in the same row with different superscripts are significantly different (*p* < 0.05).

**Table 5:** Effects of the incorporation of *Urtica urens* into broiler feed on blood analysis results (*n* = 10 per treatment).

	Treatments				
	Control	UU1	UU2	SEM	*p*-Value
Blood composition					
Cholesterol (mg/dL)					
Total	153	140	144	2.3	0.059
HDL ^1^	96.1 ^a^	88.8 ^b^	88.9 ^b^	1.41	0.046
LDL ^2^	53.5	46.8	50.4	1.20	0.072
Triacylglycerols (mg/dL)	56.7	49.6	50.3	7.65	0.760
Glucose (mg/dL)	195	212	182	22.4	0.620
Total protein (g/dL)	2.16	2.41	2.25	0.29	0.820
Albumin (g/dL)	1.04	1.19	1.08	0.18	0.840
Globulin (g/dL)	1.15	1.22	1.04	0.12	0.550
Total bilirubin (mg/dL)	0.030	0.030	0.020	0.001	0.270
Hepatic function					
AST ^3^ (IU/L)	286	357	372	61.7	0.580
ALT ^4^ (IU/L)	2.75	4.25	3.58	1.07	0.620
ALP ^5^ (IU/L)	1768	1885	1904	445.8	0.940
GGT ^6^ (IU/L)	13.5	11.5	13.5	2.03	0.727
Renal function					
Uric acid (mg/dL)	2.53	2.93	2.65	0.46	0.820

UU1- basal diet with 1% dried *Urtica urens*; UU2 - basal diet with 2% dried *Urtica urens.* ^1^ HDL—high density lipoprotein; ^2^ LDL—low density lipoprotein; ^3^ AST—aspartate transaminase; ^4^ ALT—alanine transaminase+; ^5^ ALP—alkaline phosphatase; ^6^ GGT—glutamyltransferase; SEM: standard error of the mean; mean values in the same row with different superscripts are significantly different (*p* < 0.05).

**Table 6:** Effects of the incorporation of *Urtica urens* into broiler feed on caecal VFA concentration (*n* = 10 per treatment).

		Treatments			
	Control	UU1	UU2	SEM	*p*-Value
Total VFA (mmol/mL)	8.64	9.44	9.82	0.65	0.437
Proportion (g/100 g of total VFA)					
Acetic	75.0	76.9	76.1	1.4	0.659
Propionic	6.58	6.88	6.65	0.57	0.923
Butyric	16.0	13.9	15.1	1.3	0.552
Minor	2.43	2.36	2.12	0.54	0.916

UU1—basal diet with 1% dried *Urtica urens*; UU2—basal diet with 2% dried *Urtica urens.* VFA = volatile fatty acids; SEM: standard error of the mean.

**Table 7:** Effects of the incorporation of *Urtica urens* into broiler feed on ileum villi development (*n* = 10 per treatment).

		Treatments			
	Control	UU1	UU2	SEM	*p*-Value
Height (µm)	805	874	802	61.8	0.651
Width (µm)	103	106	99.0	3.9	0.418
Depth (µm)	149	134	123	12.6	0.361
Absorption area (mm^2^)	0.26	0.29	0.25	0.02	0.378

UU1—basal diet with 1% dried *Urtica urens*; UU2—basal diet with 2% dried *Urtica urens.* SEM: standard error of the mean.

## Data Availability

The data are contained within the article.
